# Using Endoscopic Optical Coherence Tomography to Detect and Treat Early-Stage Pancreatic Cancers

**DOI:** 10.3389/fonc.2021.591484

**Published:** 2021-03-15

**Authors:** Lanchun Lu, Zhilin Hu, Wendy Frankel, Rulong Shen, Wei Chen, Xueliang Pan, John C. Grecula, Mark P. Bloomston, Mary E. Dillhoff

**Affiliations:** ^1^ Department of Radiation Oncology, The James Cancer Hospital and Solove Research Institute, Wexner Medical Center and College of Medicine at the Ohio State University, Columbus, OH, United States; ^2^ Pharos Scientific, LLC, Lilburn, GA, United States; ^3^ Department of Pathology, The James Cancer Hospital and Solove Research Institute, Wexner Medical Center and College of Medicine at the Ohio State University, Columbus, OH, United States; ^4^ Department of Biomedical Informatics, College of Medicine at the Ohio State University, Columbus, OH, United States; ^5^ 21st Century Oncology, Fort Myers, FL, United States; ^6^ Department of Surgical Oncology, The James Cancer Hospital and Solove Research Institute, Wexner Medical Center and College of Medicine at the Ohio State University, Columbus, OH, United States

**Keywords:** endoscopic optical coherence tomography, early-stage pancreatic cancer, detect and treat, endo-optical coherence tomography–guided brachytherapy, high-dose rate brachytherapy

## Abstract

We developed a novel technology capable of detecting early-stage pancreatic cancers using high-resolution three-dimensional endoscopic optical coherence tomography (Endo-OCT), and treating them using high dose rate brachytherapy (HDR) under the Endo-OCT image guidance. This technology integrates our custom-built ultra-high resolution endoscopic three-dimensional OCT diagnostic imaging device with a commercial high dose rate brachytherapy system (HDR), resulting in a compact, portable, easy-to-operate, and low-cost Endo-OCT image-guided high dose rate brachytherapy (OCT-IGHDR) system. The system has the dual functions of diagnosis and treatment that can precisely detect and measure the location and size of the early-stage pancreatic cancer or premalignant lesions and then treat them from the inside of the pancreatic duct with an accurate and focused dose while greatly reducing the radiation toxicity to the neighboring tissues and organs. This minimally-invasive treatment technology could avoid the potential complications from surgery and reduces the high operation cost. This technology could also be applied to treat diseases of the esophagus, rectum, bronchus, and other aerodigestive organs that are suitable for use with an endoscopic device. In this article, we describe the concept of this technology and the preliminary experiments that could demonstrate the concept by using this homemade Endo-OCT machine to image the pancreatic duct for diagnosis of early-stage pancreatic cancer or premalignant lesions and to perform Endo-OCT image-guided brachytherapy.

## Introduction

### Reason for Early Detection of Pancreatic Cancer

**Figure 1:**
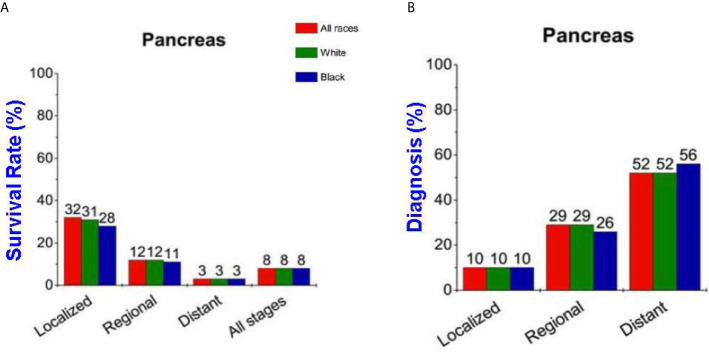
**(A)** Pancreatic cancer survival rate strongly depends on the stage of disease. **(B)** Percentage of patients diagnosed versus the stage when diagnosed.

More than 57,600 estimated new pancreatic cancer cases were diagnosed in the United States in 2020, and the estimated deaths are more than 47,050 (1). The 5-year overall survival rate for pancreatic cancer is 9% and on average the survival time is less than 12 months after diagnosis (2). The 5-year survival rate strongly links to the disease stages at diagnosis thus making it important to develop methods to aid in earlier diagnosis (**Figures 1A, B**) (1). The poor survival rate is mainly due to the late diagnosis of the cancer which advances very aggressively leaving few options for treatment. Early detection and treatment of the disease is the key to prolonging patients’ lives but in current clinical practice there are still no satisfactory techniques available for early detection.

### Why We Need Endoscopic OCT Imaging

Major research efforts in early detection of pancreatic cancer have come from different avenues, from new biomarker identification to imaging modalities. For example, a screening method to detect early-stage pancreatic cancer was recently developed by Melo et al. (3) and L.D. Mellby et al. (4). No biomarker has been found to reliably diagnose pancreatic cancer and only Ca 19-9 is used clinically. Biomarkers also lack the ability to localize the cancer without the assistance of imaging. Most of the imaging modalities such as MRI, CT, PET-CT, or ultrasound have difficulty detecting small, early-stage or premalignant lesions. A recently developed imaging technique, Optical Coherence Tomography (OCT), has spatial resolution on the level of micrometers (µm) and thus may play a role in the early detection of pancreatic cancer compared to traditional cross-sectional imaging.

OCT is an imaging modality that was first developed by D. Huang et al. in the early 1990s (5, 6). It obtains images of the object of interest by reconstructing profiles of the optical coherence between the lights from the reference arm and the lights from the sample arm that are originally split from the same light source (usually a near-infrared light source). Lights from the sample arm irradiate the object to be imaged and then are refracted and scattered. A spectrometer collects the coherence signals, the interferences between the lights from the reference arm and the lights from the sample arm, and sends the coherence signals to the computer to analyze and reconstruct the images. The spatial resolution of OCT image is at the µm level. The current applications of OCT are mostly focused on the imaging of biological tissues, especially of the human eyes (7, 8), skin (9), and cardiovascular tissue (10, 11) while the clinical practice of using OCT for cancer detection is still uncommon, especially for pancreatic cancer.

**Figure 2:**
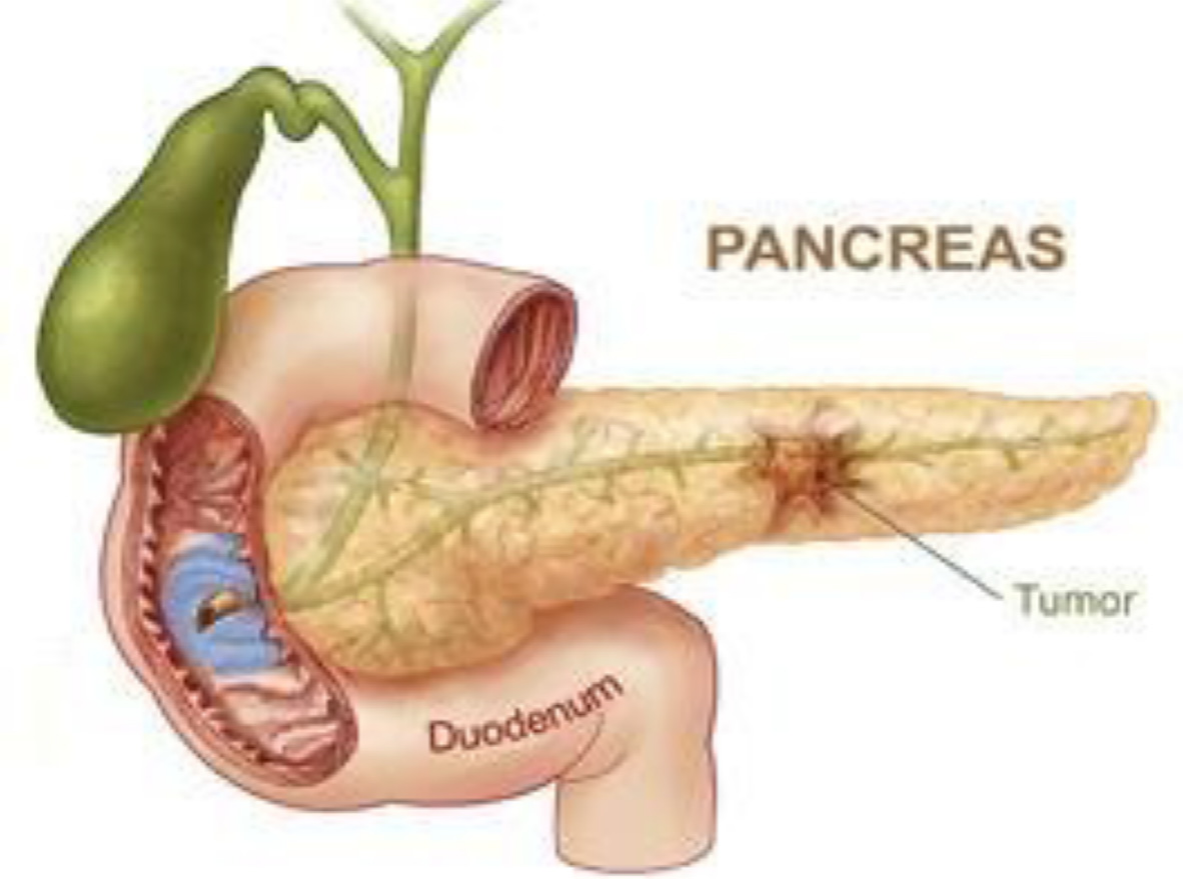
Cartoon of ductal pancreatic cancer.

Considering that about 90% of pancreatic cancers initially arise from the epithelial lining of pancreatic ducts (12, 13) (**Figure 2**), we believe that the endoscopic imaging approach may be an improved method for early detection of pancreatic cancers (14, 15). Endoscopic imaging has been widely used in the field of medicine. Traditional endoscopy uses endoscopic cameras to photograph disease areas by placing the endoscopic camera inside the aerodigestive organs such as esophagus, trachea and bronchi, intestines, rectum, and stomach. However, the images obtained by traditional endoscopy can only see the surface of these organs and lack the ability to detect abnormalities that may occur under the mucosa and not readily visible to the eye. Endoscopic ultrasound (EUS) combines the technology of endoscopy and ultrasound to obtain images of the digestive tract and has been applied in clinical practice for imaging pancreatic cancer. However, the spatial resolution of EUS is much less than that of OCT (millimeter vs. µm), thus an endoscopic OCT will be complementary and possibly superior in detecting early-stage cancers or premalignant lesions. Based on this idea we need to build an endoscopic type and catheter-based OCT (Endo-OCT) for early diagnosis of pancreatic cancer.

### Reason for Endo-OCT Guided Brachytherapy

Early diagnosis of pancreatic cancer will be followed by its treatment. Currently the only curative treatment for pancreatic cancer is surgery. Although surgery for pancreatic cancer has become much safer over the last several decades it still carries significant morbidity and mortality. With an imaging modality that has the ability to detect earlier disease local treatments that preserve the pancreas could be very useful. Radiation therapy may be one such good option for local treatment.

Radiation therapy has been successful in treating many types of cancers but its utility in pancreatic cancer has been debated (16–19). Many clinical trials of radiation therapy on pancreatic cancer are based on image-guided external beam radiation therapy (EBRT): 3D conformal radiation therapy, Stereotactic Body Radiation Therapy (SBRT), and Intensity Modulate Radiation Therapy (IMRT) (20–23). The results of these previous trials are mixed, with some showing increased survival but also with severe toxicity related to the radiation. The main problem with EBRT is that radiation beams have to pass through healthy critical organs surrounding the pancreas before they can reach and deposit radiation on the tumors inside the pancreas. Pancreatic cancer is also more radiation -resistant compared with other types of tumors, requiring higher radiation doses to kill the tumor cells. Strong evidence has indicated that a focused high radiation dose to the disease site can greatly improve the treatment efficacy (24). Higher dose to the tumor will result in higher toxicity to the surrounding healthy critical organs. In addition, precisely targeting small pancreatic cancers with EBRT has remained difficult due to organ motion and the poor resolution of the conventional image modalities (CT, ultrasound, and MRI). Endo-OCT may help solve some of the current clinical problems in treating patients with pancreatic cancer at the early stages without going through surgery to remove or partially remove the pancreas. The endoscopic setup allows for possible delivery of a radioactive source to the location of the tumor to perform a localized brachytherapy with a highly focused radiation dose to the tumor through the catheter after imaging for diagnosis while minimizing the dose, and hence toxicity, to the surrounding normal tissues/organs.

Here as a proof of concept, we report the Endo-OCT technology that we have been developing not only for the early diagnosis of early-stage pancreatic cancers or premalignant lesions such as Intraductal Papillary Mucinous Neoplasms of the pancreas (IPMNs) (25), but also for the treatment of them with the Endo-OCT image-guided HDR brachytherapy (Endo-OCT IGHDR). We will first describe our custom-built 3-D endoscopic OCT imaging device dedicated to diagnostic applications, then the proposed technology to integrate the Endo-OCT imaging device to perform Endo-OCT IGHDR (26). We will also present the preliminary experiments and results to demonstrate the feasibility of this technology.

## Methods and Materials

Using our proposed technology and the funding supported by Pelotonia Idea Grant Award (15), we built an ultra-high-resolution three-dimensional endoscopic OCT imaging device to possess the dual functions of 1) detection of early-stage pancreatic cancer or premalignant lesions and 2) OCT-guided HDR brachytherapy for such lesions.

### The Physics of Optical Coherence Tomography

**Figure 3:**
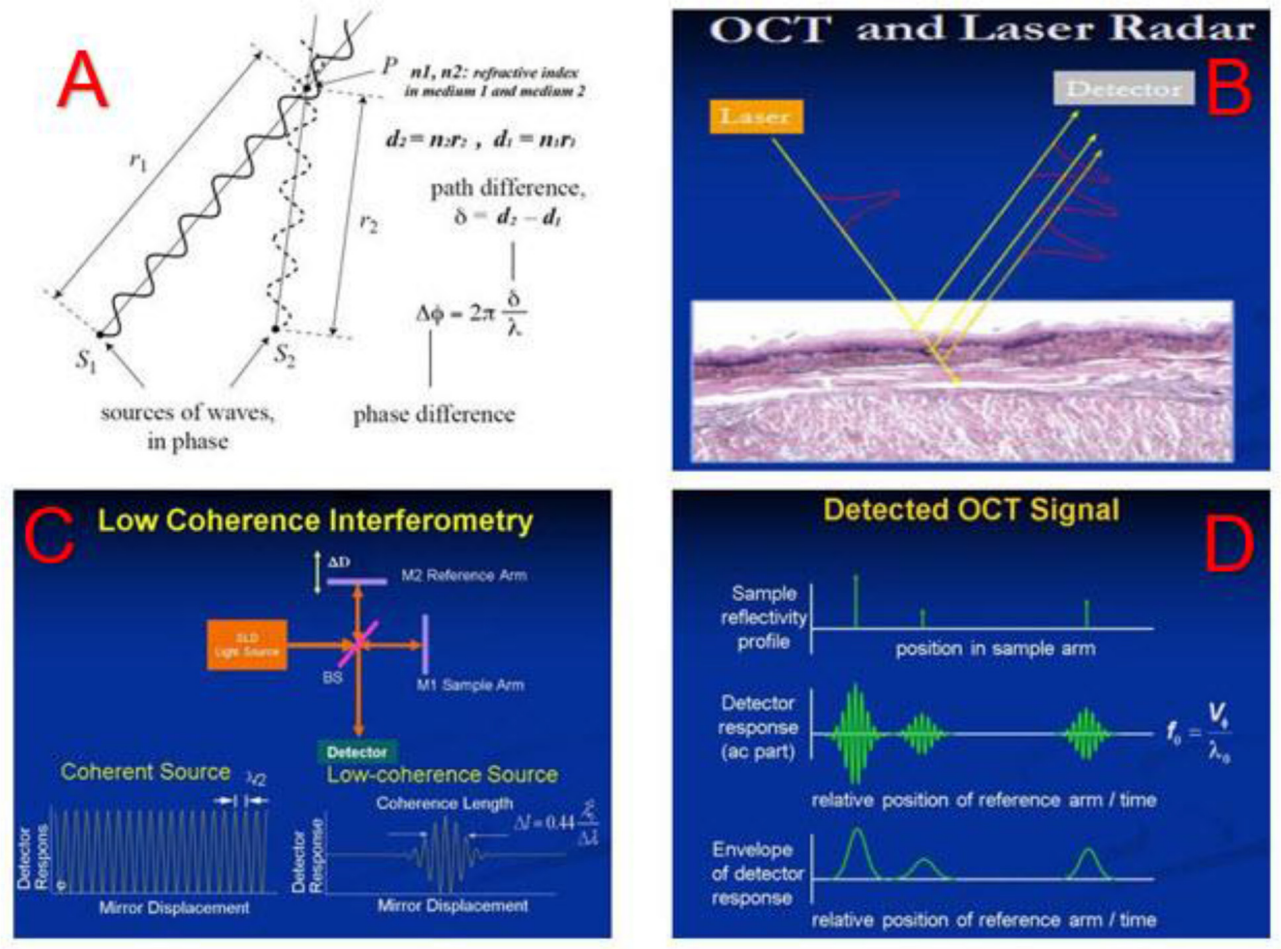
The physics principle of Optical Coherence Tomography (OCT). Top left **(A)** The optical path difference and the phase difference between the incoming and the refracted lights. Top right **(B)** The incoming laser light interacting with tissues and being refracted and collected by a detector. Bottom left **(C)** the optical coherence interference generated in the OCT system. Bottom right **(D)** the OCT signal being formed and detected.

The physics principles of OCT are straightforward. When two coherence optical waves meet and overlay, at a point of interest where the detector measures if the optical path difference between these two waves is close to zero or integer numbers of the wave length, there will be interference fringes on the amplitude of the composed wave. **Figure 3A** shows incoming sources of optical wave interfering with the refractive optical wave and the phase difference between them inducing the coherence of these two waves. **Figure 3B** shows the incoming laser light penetrating into tissues and being refracted to reach the detector. The phase difference between the incoming and refracted lights will determine the amplitude of the coherence between them. **Figure 3C** depicts how the OCT system creates a coherence interference from the incoming light and the refractive light: the Superluminescent Diode (SLED) emits a low coherence light, and the light is split into the reference arm and the sample arm. The light in the reference arm is directly reflected by a mirror (M2) while the light in the sample arm goes into and is refracted by the tissue to be imaged before reflected by another mirror (M1). The tissue’s refractive index and the depth of the light going through determine the optical path length in the sample arm and hence the path difference between the sample arm and the reference arm, which compose a spectrum of optical interference that is associated with the density and structure of the tissue (**Figure 3D**). The interference signals are collected by the OCT detector. By reconstructing the spectrum of the optical interference using Fast Fourier Transform (FFT) technology (27), we obtain the images of the tissue.

### Endoscopic OCT Imaging System

**Figure 4:**
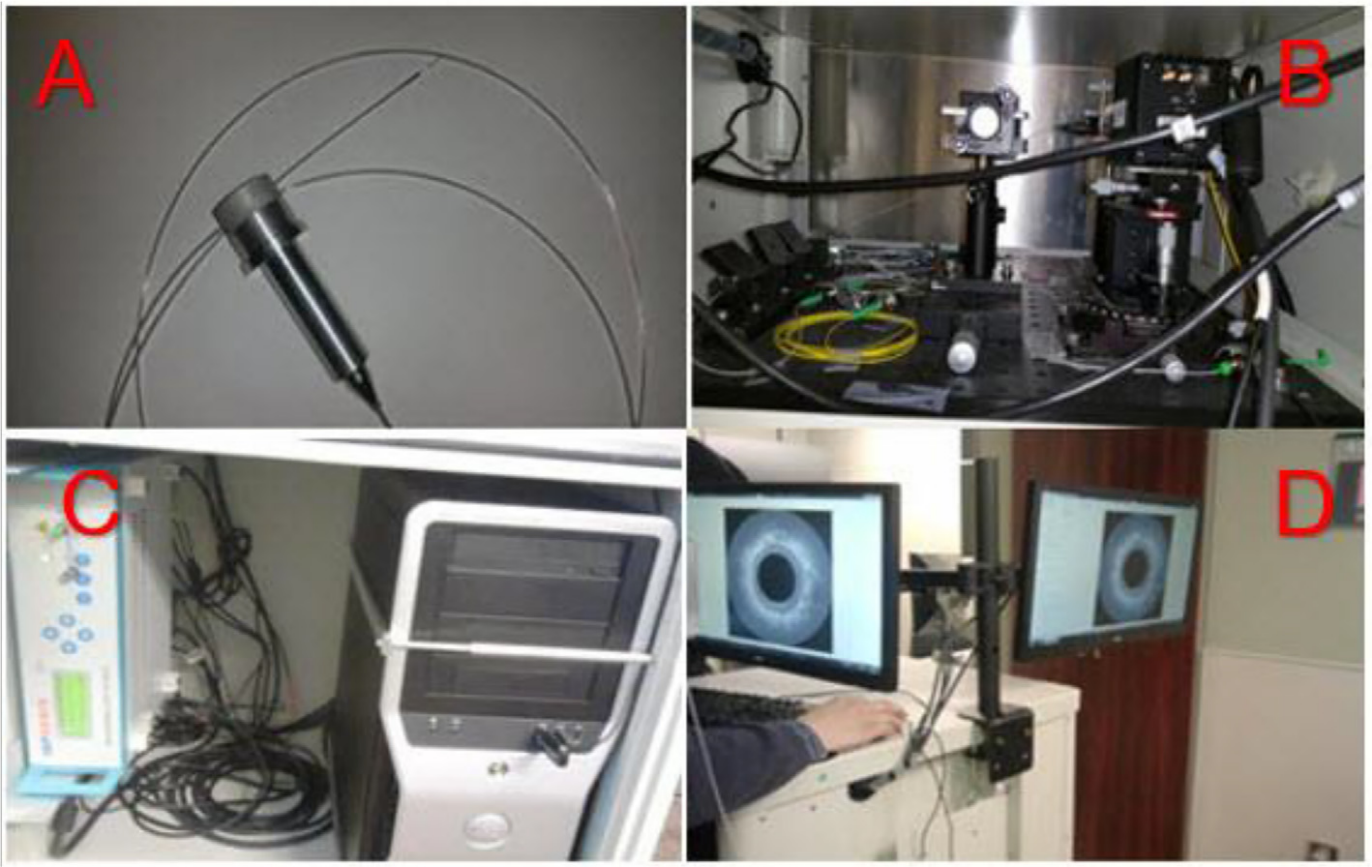
Our custom-built 3D ultra-high resolution OCT system. Top left **(A)**: OCT probe (detector, optical fiber, and catheter). Top right **(B)**: OCT spectrometer hardware system. Bottom left **(C)**: OCT light source and raw data process system. Bottom right **(D)**: OCT image display system.

**Figure 5:**
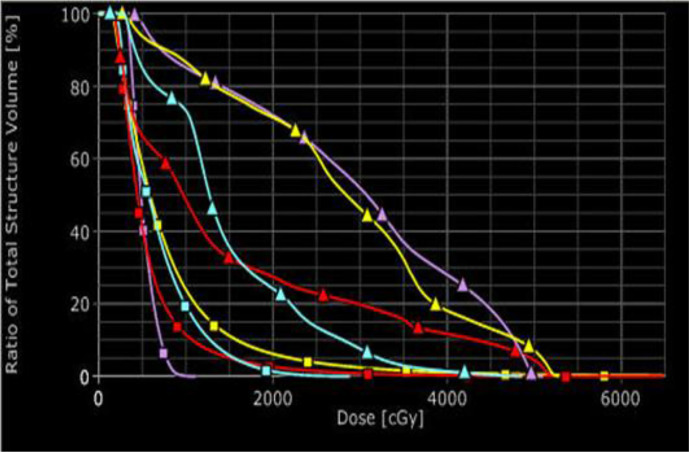
Comparison of dose volume histograms for the organs at risk for EBRT (triangles) and HDR (squares): sky-blue for kidney, red for liver, purple for stomach, and yellow for bowel.

The 3-dimensional (3D) ultra-high-resolution non-invasive Endo-OCT imaging device includes five components as shown in **Figure 4**: (1) the OCT probe that includes the OCT detector (diameter: 0.8 mm, length: 1.2 mm) attached to an optical fiber that transfers the illuminating light and signal between the source and the receiving optical spectrometer, **Figure 4A**; (2) broad bandwidth NIR light source, **Figure 4C**; (3) the soft and transparent OCT catheter tube (inner diameter: 1 mm; outer diameter: 2 mm) that allows the OCT probe to move through to the disease site for imaging, **Figure 4A**; (4) the optical spectrometer that acquires optical information with optimum imaging depth, **Figure 4B**; (5) the console computer that processes the signals, reconstructs raw data into images, displays real-time or offline OCT images, and controls the operation of the imaging system, **Figures 4C, D**.

The OCT probe (**Figure 4A**) is composed of an OCT detector/camera that attaches to the tip of an optical fiber which moves inside a soft and flexible transparent catheter to the region of interest to image. When the Endo-OCT system is in operation, first the catheter is inserted into the patient’s pancreatic duct. Then the OCT detector/camera attached to the optical fiber slides inside the catheter. The other end of the optical fiber connects to the optical system. When imaging, the OCT detector is pulled back by set intervals. At each interval the detector rotates 360° to take a full image in the radial direction, a slice corresponding to that step which is essentially the longitudinal position in the duct. The pullback and rotation of the detector are operated through a programmed motor controlled by the computer of the Endo-OCT system. Images of all the slices together will compose the 3D image of the pancreatic duct. **Figure 4B** shows the spectrometer composed of mirrors and prisms. The interference of optical signals collected by the detector is processed through the spectrometer (**Figure 4B**) and then is reconstructed into images using the computer shown in **Figure 4C**. The figure on the bottom right (**Figure 4D**) shows the two computer monitors that display synchronously the live OCT images while the OCT detector moves through the pancreatic duct to image. The monitors can also display offline images saved from previous scans.

When imaging, the 0.8 mm diameter OCT detector attached to the flexible optical fiber is inserted through the side port of an endoscope and into the human pancreatic duct to acquire high-resolution tomographic cross-sectional 3D images of biological tissue microstructure *in vivo* and in real-time. The time to acquire the full duct’s images is only a few minutes depending on the intervals and the speed of the pullback. The imaging rate is 91,911 lines per second as determined by the line scan camera, 1024-LDH2 92KHz InGaAs, equipped in the system.

To make sure the system worked properly, we first used it to image a quality assurance (QA) phantom—a simple small device that we fabricated for testing the imaging ability and functionality of the Endo-OCT system. After the phantom test passed and we confirmed the Endo-OCT imaging system worked on the phantom, we used the imaging system to perform some clinical studies on evaluating the pancreatic duct in resected pancreas specimens.

### Endo-OCT Guided Brachytherapy of Pancreatic Cancer

After early pancreatic cancer is detected and located, the next step is to treat it. Brachytherapy, also known as internal radiotherapy, treats a patient by placing a radiation source inside or next to the tumor. This allows delivery of much higher focused doses to the pancreatic cancer while keeping the organs at risk (OARs) at significantly low toxicity. We had performed a simulation of treatments with HDR brachytherapy and EBRT and compared the toxicity to OARs from HDR and EBRT. The results of the simulation study showed the superiority of brachytherapy over EBRT in terms of the toxicity to OARs while delivering the same dose to tumors (**Figure 5**). There was a study using low dose rate brachytherapy (LDR) to treat advanced- and late- stage pancreatic cancers, where low dose rate radioactive sources were implanted inside the pancreatic duct and tumors (28). However, LDR is a complicated and time-consuming invasive procedure. Its treatment time is long, varying from several days for most cancers to up to months for treatments like permanent seed-implants for prostate cancer. Unlike LDR, HDR allows the radioactive source to move easily into and out of the treatment site which is controlled by a computer. Because of this, HDR has become popular in radiation therapy due to its easy delivery and the short treatment time.

In HDR brachytherapy, the source delivery through a transparent catheter tube to the treatment site to treat a tumor is very similar to the Endo-OCT imaging system sending the OCT camera through a transparent catheter tube to the disease site to image the region of interest. Because of this feature we adapted an existing commercially available HDR system (GammaMedplus iX, Varian Medical Systems, Inc. Palo Alto, California) to deliver the high-rate radioactive source to treat lesions, and integrated it into the Endo-OCT imaging system to create the Endo-OCT image-guided high dose rate brachytherapy system (26). The HDR was easily coupled to our Endo-OCT imaging system without needing any modifications to the HDR system itself. The commercial catheter used as a source tube in the HDR system is the same in diameter as the one we used in the Endo-OCT system to send the OCT detector in for imaging. This means that either the OCT detector or radioactive source can move inside the source catheter of HDR. However, the commercial HDR source catheter is produced and provided by the manufacturer of the HDR machine, which is specifically used in clinic for HDR brachytherapy. Its connector only works for the HDR machine and can only be plugged into the source channels of the HDR machine to deliver the radioactive source for treatments. It cannot be directly plugged into the Endo-OCT machine or connected to the OCT imaging catheter. We built an OCT-Brachy connector (OBC) to use it as a switch to connect the catheter inside the pancreatic duct to the Endo-OCT system for imaging and to connect the catheter to the HDR system for HDR brachytherapy. The catheter inserted inside the patient’s pancreatic duct to hold the OCT probe for imaging is the same pathway used to deliver the radiation source to the target area. The OCT-Brachy connector allows the system to transition from diagnosis mode to treatment mode easily and promptly.

**Figure 6:**
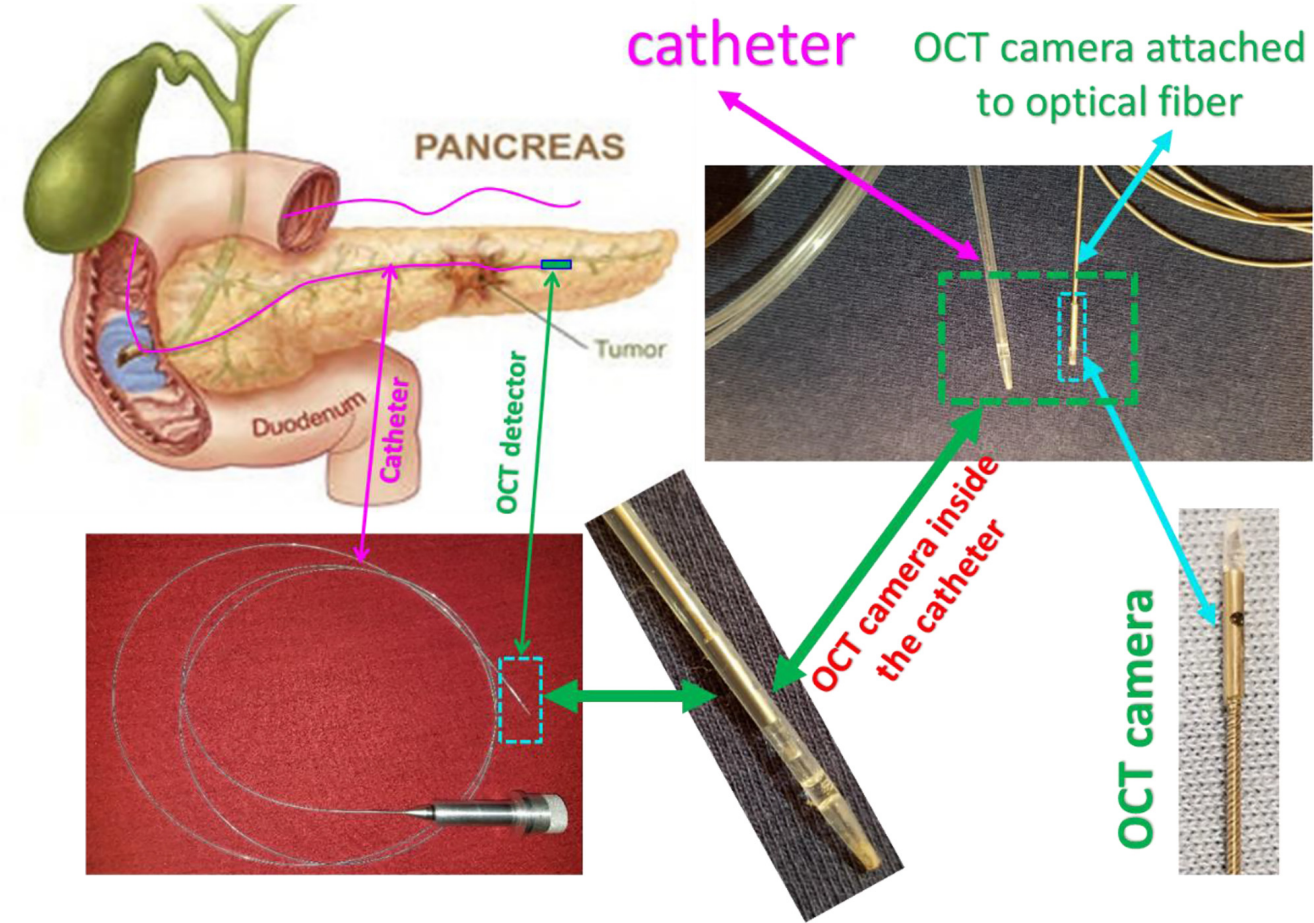
The scheme of OCT-guided HDR brachytherapy (OCT-IGHDR) and the photos of the OCT probe and the catheter.

**Figure 7:**
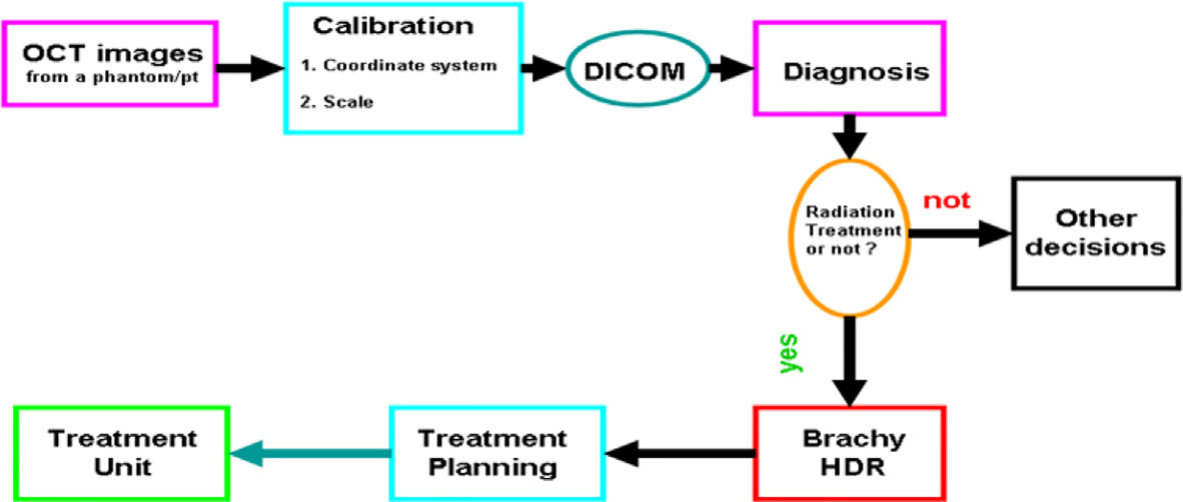
The flow chart for OCT-guided HDR brachytherapy system (OCT-IGHDR).

**Figure 8:**
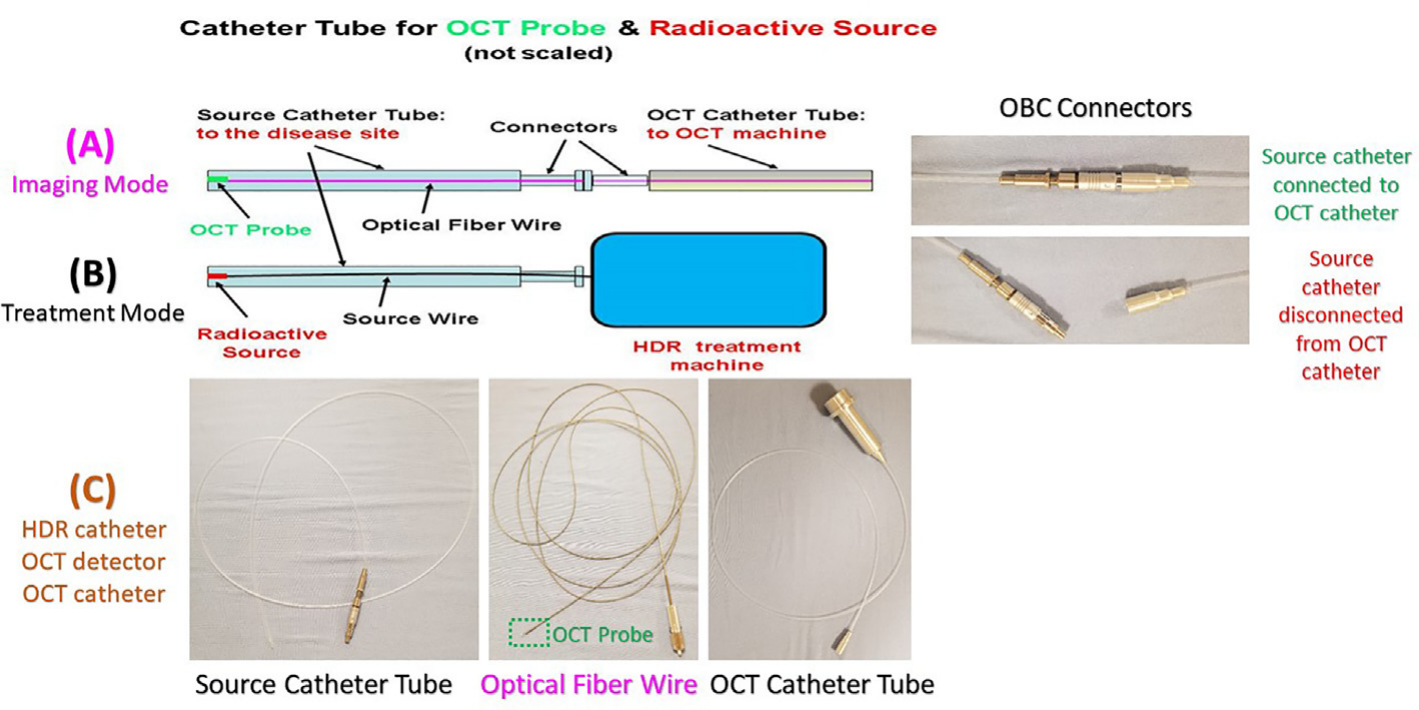
The OCT imaging catheter tube, OCT-Brachy Connector (OBC), source catheter tube, and the display of the switch between the imaging mode and the treatment mode. Top row **(A) ** for Imaging Mode, middle row ** (B) ** for Treatment Mode, and bottom row ** (C) ** for HDR catheter, OCT detector, OCT catheter.

**Figure 6** depicts a cartoon of the anatomical structure of a pancreas and how a catheter is placed inside the pancreatic duct that allows the Endo-OCT detector to go through for imaging as well as the HDR source to be delivered for Endo-OCT IGHDR. In **Figure 6**, beside the cartoon are the photos of the OCT camera attached to the optical fiber, the catheter, and the camera inserted inside the catheter. **Figure 7** shows the schematic work flow chart of the Endo-OCT IGHDR system, and **Figures 8A, B** displays how to switch between the imaging mode and the treatment mode when the Endo-OCT IGHDR system is in operation. Photos in **Figure 8C** show the source catheter tube, the OCT detector/camera attached to the optical fiber wire, the OCT catheter tube, and the OBC connector that we built and used for the Endo-OCT IGHDR system.

As shown in **Figure 7**, imaging the pancreatic duct to detect the cancer is first, and then treating the cancer after it is diagnosed and located. Before acquiring OCT images, the source catheter tube is first inserted inside the patient’s pancreatic duct. Then the OBC connector connects the source catheter tube to the OCT catheter tube that attaches the Endo-OCT imaging system (**Figure 8A**). To acquire images, the Endo-OCT detector moves from the OCT system through the endoscopic OCT catheter tube, passes the OBC, goes into the source catheter tube and reaches the region of interest (ROI) to start imaging. While imaging the ROI, the detector is being pulled back at a preset interval and the detector scans 360 degrees to collect raw imaging data. The image system is calibrated so that the Endo-OCT images have correct coordinates and scale for the ROI being scanned. The reconstructed images are then converted into DICOM format and sent to diagnosis unit for confirmation with a pathologic result and to the HDR treatment planning system for treatment planning if physicians decide to treat the lesion.

**Figure 9:**
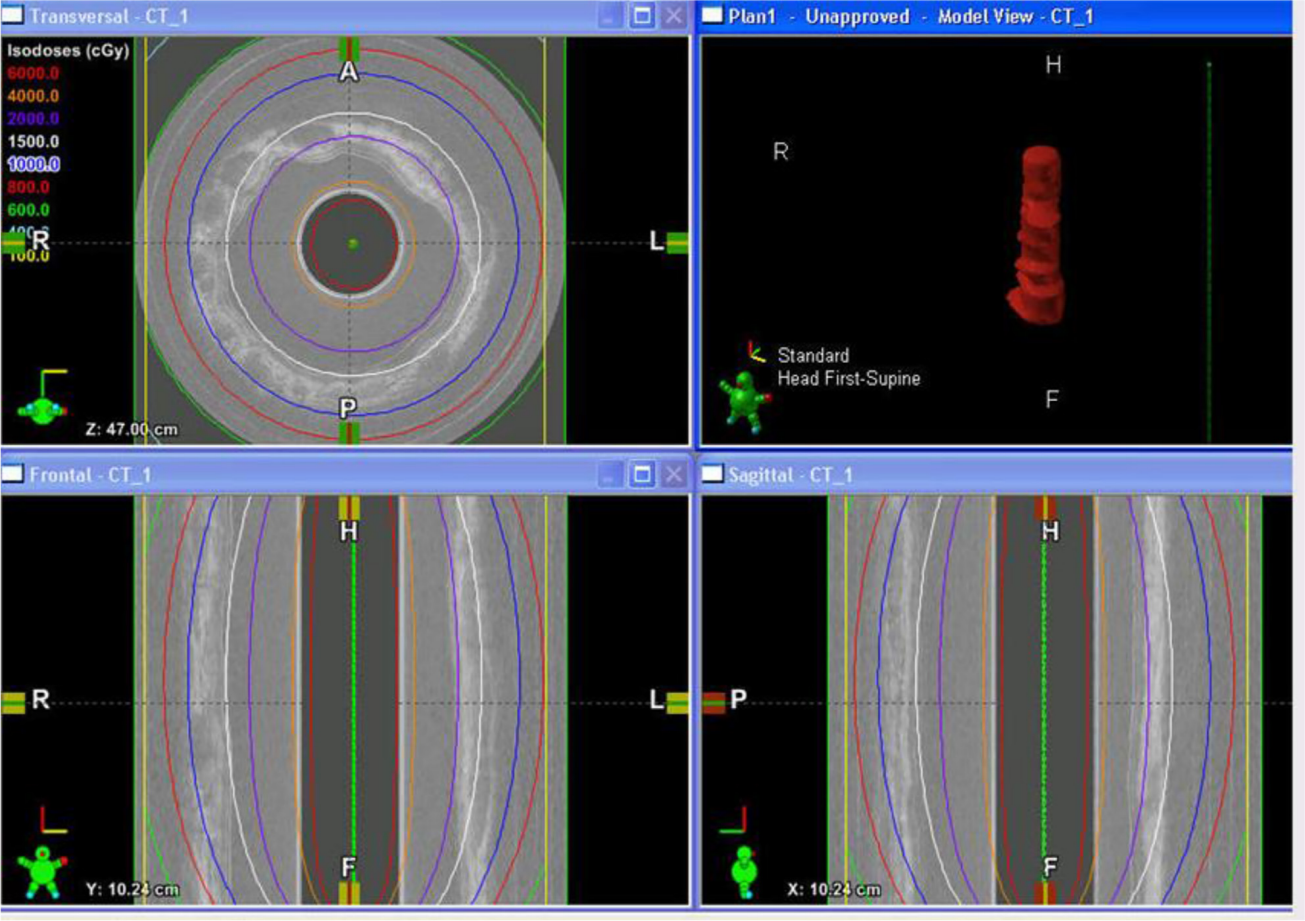
The display of an example of the OCT-guided HDR brachytherapy (OCT-IGHDR) treatment plan and the dose distribution.

To treat the early-stage lesions, the Endo-OCT imaging system is first disconnected from the OBC connector while the source catheter tube is left inside the patient’s pancreatic duct. The process is demonstrated in the cartoon of **Figure 8**. Then the source catheter is connected to the HDR system through the OBC connector to deliver the radiation source to the target area (**Figure 8B**). Before delivering an Endo-OCT IGHDR treatment, we need to perform a treatment simulation, referred to as treatment planning, in the treatment plan computer by using the acquired diagnostic OCT images of the lesion. The treatment plan determines how much dose the lesion needs to receive, where the source is delivered to and how long the source will stay in position. **Figure 9** shows an example of treatment planning based on a set of Endo-OCT images using Varian’s BrachyVision software. According to the treatment plan, the computer-controlled HDR treatment unit knows exactly where to deliver the radiation source and how long it will stay there to treat the lesion according to the prescribed dose. With the Endo-OCT IGHDR system we are able to deliver an Endo-OCT guided radiation therapy for early-stage pancreatic cancer, just like what we do for other HDR treatments nowadays in clinic.

## Results

### Imaging on the QA Phantom to Test the Endoscopic OCT System

**Figure 10:**
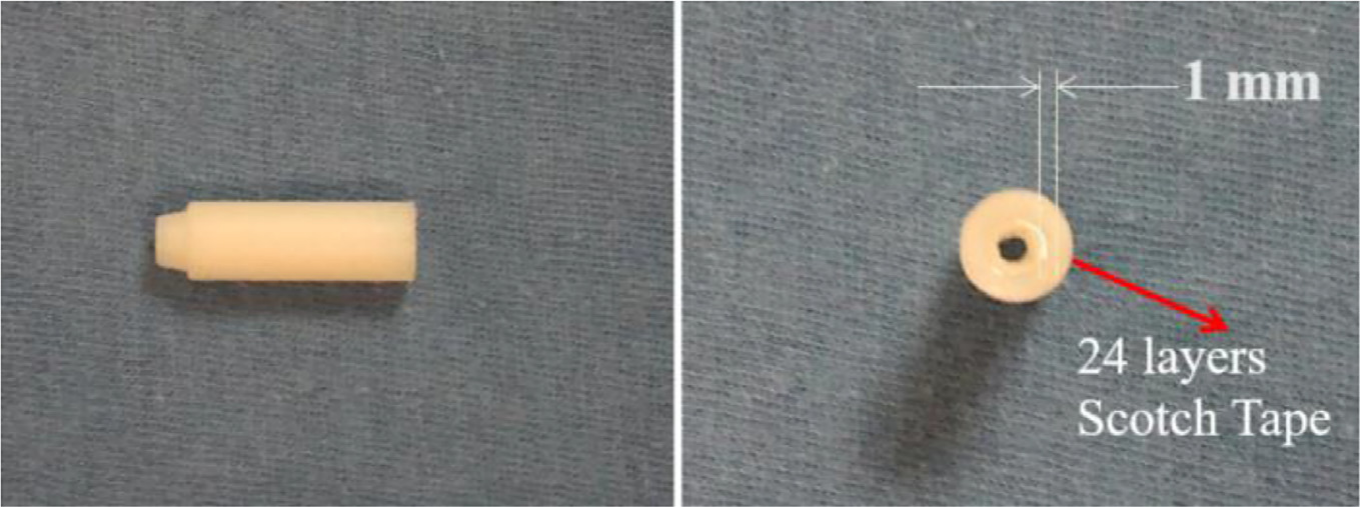
The Endo-OCT test phantom—Scotch Magic Tape phantom.

**Figure 11:**
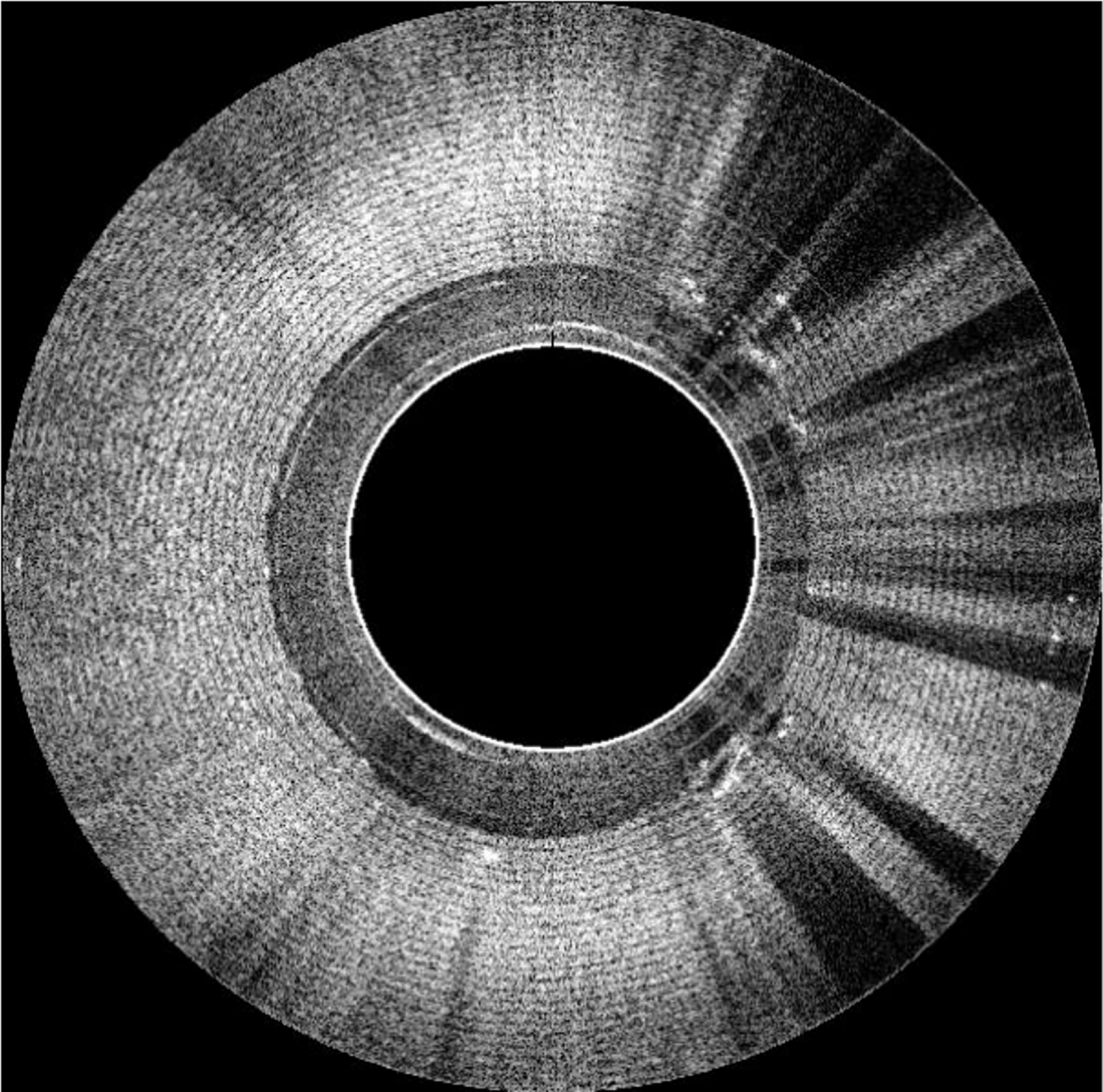
The OCT image of the Endo-OCT test phantom—Scotch Magic Tape phantom.

We made a small, simple phantom that can be used to perform the QA for the Endo-OCT imaging system. This phantom is a wrap of 24 layers of Scotch Magic tape to mimic a pancreatic duct or any tubular organs (**Figure 10**). The axial view of the OCT image of this phantom acquired using the Endo-OCT imaging system is shown in **Figure 11**. The thin layers of Scotch Magic tape are clearly seen in the image.

### Imaging on Patients’ Resected Pancreas Using Endo-OCT System

**Figure 12:**
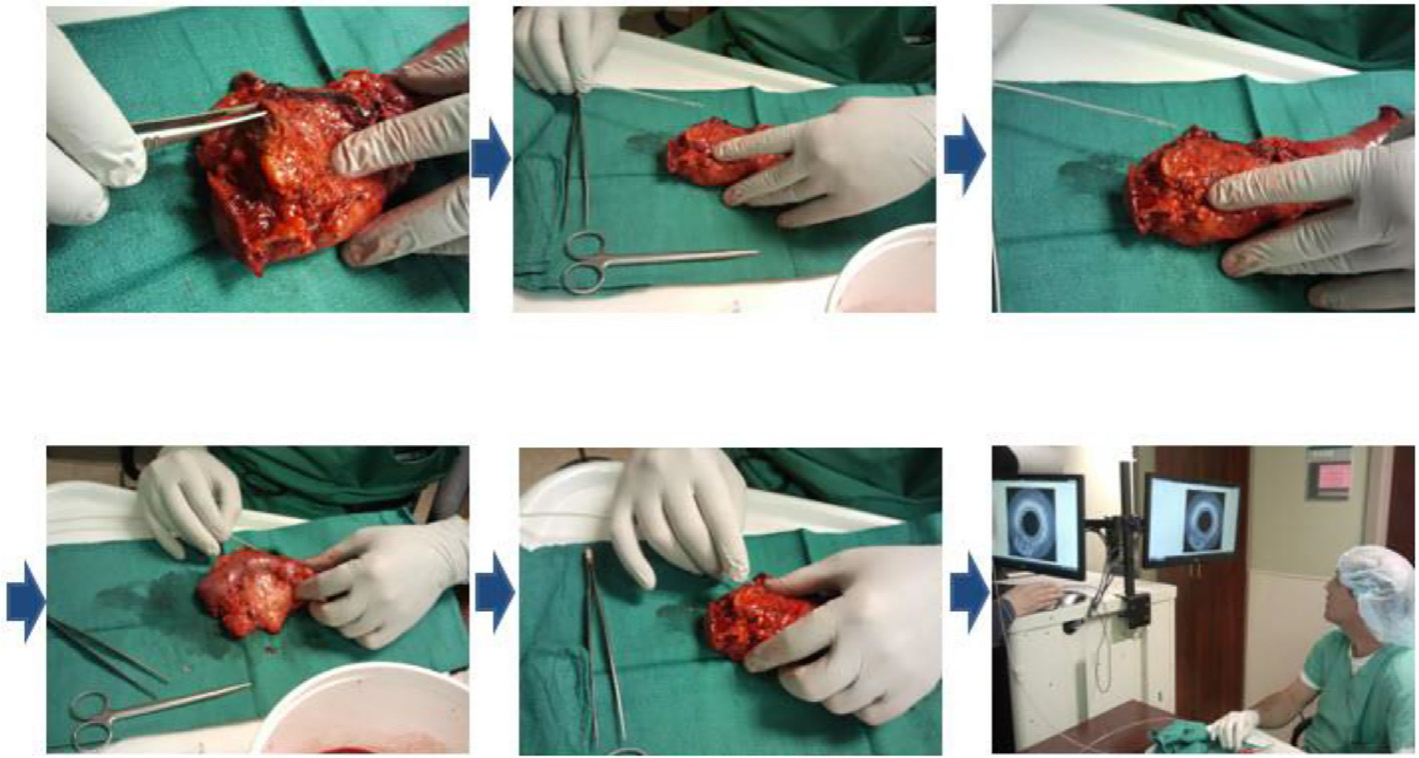
OCT imaging on a human patient’s resected pancreas (specimen).

**Figure 13:**
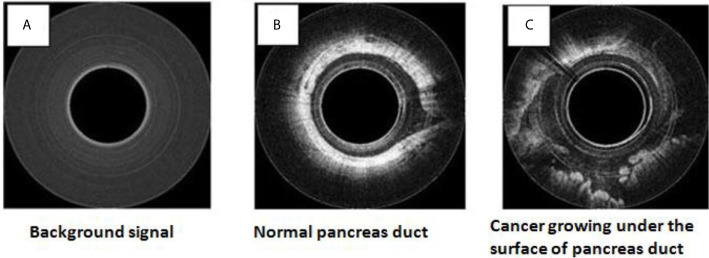
OCT images for the human patient’s resected pancreas (specimen): background **(A)**, normal duct region **(B)**, cancerous region **(C)**.

**Figure 14:**
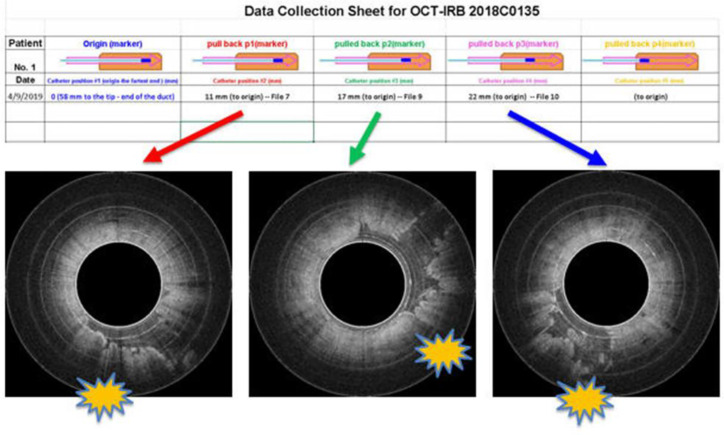
Endo-OCT images show three abnormal structures appearing at three locations underneath the surface of a patient’s pancreatic duct.

To safely evaluate the ability of the Endo-OCT system to image the pancreas a pre-clinical trial was performed using resected pancreas specimens after they were removed from the patient at surgery. IRB approval was obtained at the Ohio State University. To operate the machine and use it to image a pancreas duct, we first inserted a transparent catheter tube into the pancreas duct. Then the OCT detector moved through inside the catheter to the region of interest and imaged the area. Photos in **Figure 12** demonstrate the process of this experiment. The Endo-OCT device was manipulated to image the pancreatic duct and to detect abnormalities in the ducts in patients undergoing surgery. The catheter’s inside diameter is 1* mm*, outside diameter 2* mm*, which allows the computer-controlled 0.8* mm* diameter OCT detector to move through inside the catheter to take images of the pancreatic duct. The imaging results of background (no signal, **Figure 13A**), normal region (white uniform area indicates a normal duct wall, **Figure 13B**), and cancerous region (white protuberant area indicates an abnormal structure inside the duct wall, **Figure 13C**) of the pancreatic duct is shown in **Figure 13**. The diagnostic result from the imaging was later confirmed by the pathologic and biopsy examinations. **Figure 14** shows another patient’s clinical trial result, where there are three abnormal structures appearing at three different locations underneath the surface of the pancreatic duct. For this case, the duct wall had been invaded by tumor cells.

### Progress on Endo-OCT Guided Brachytherapy of Pancreatic Cancer 

Based on the concept and design of our Endo-OCT guided radiation therapy (15, 26), we have successfully built major parts such as the OBC connector to connect the endoscopic OCT imaging catheter for imaging and the source catheter for HDR treatment (**Figure 8**). We tested and verified this work flow in dry runs using a phantom by following the work flow showed in **Figures 7** and **8**, starting from imaging to the completion of HDR brachytherapy. It worked successfully and smoothly.

## Discussion

In this paper, we present a promising catheter-based application of endoscopic OCT to aid the early detection and treatment of pancreatic cancer or premalignant lesions. This application of using OCT on pancreatic cancer is significant as pancreatic cancer is one of the most lethal cancers that would benefit from early diagnosis and treatment. Conventional cross-sectional imaging has not been successful in the detection of early-stage cancer, thus the use of OCT could be an important addition. Given that most pancreatic cancers arise in the luminal epithelium of the pancreatic duct, a machine that could identify abnormalities in the duct down to the µm level would aid the early diagnosis and then possibly treatment of such cancers *via* focused radiation would be beneficial to the patients.

From the diagnostic side, we have successfully built the Endo-OCT imaging machine and used it on the first clinical trial on resected human pancreas and confirmed its ability to detect abnormalities in the pancreatic duct. Traditional endoscopy visualizes the surface of organs or the lumen of the duct at best, even with newer methods such as SpyGlass (29) or CellVizio (30). The Endo-OCT device is able to penetrate and image up to 3 mm deep into tissues depending on the density, and give a 3D image of the object with a resolution of ~7 µm. This resolution is hundredfold better than other standard imaging modalities in clinic. Using this µm-level spatial resolution imaging device we can detect precancerous lesions or early-stage cancers developing along the pancreatic duct as small as the µm-level in size. By studying the morphology of the observed lesions and analyzing brushings and biopsies collected with this tool, early diagnosis of pancreatic cancer becomes feasible. A clinical trial using this homemade Endo-OCT to image surgically resected pancreas with high risk pancreatic cancer (31) is ongoing.

We have also proposed and tested the idea of integrating the Endo-OCT imaging system with the commercial HDR system to compose an Endo-OCT image-guided radiation therapy system, Endo-OCT IGHDR, to treat early-stage cancers from within the pancreatic duct. This preliminary research confirms the possible utility of this novel imaging device. The feature of this technology is that the catheter tube used for Endo-OCT imaging stays temporarily inside the patient’s body and plays a role as the common pathway to deliver a localized treatment such as brachytherapy that focuses on the discovered lesion. When the Endo-OCT system is used for diagnostic imaging, the connector OBC is used as a bridge to connect the OCT imaging catheter to the source catheter tube that has already been inserted into the pancreatic duct. The OCT detector moves starting from the OCT catheter and passes through the bridge connector to the source catheter then arrives at the possible disease site to take the OCT images. As soon as the cancer is found, located, and decided to be treated with radiation therapy, the Endo-OCT detector will be pulled out from the source catheter. Then the Endo-OCT catheter is disconnected from the source catheter which will still stay at the original position for use in treatments. After the treatment plan is completed by using the acquired Endo-OCT images, the source catheter is connected to the HDR unit to perform the treatment. Switching from the diagnosis mode to the treatment mode is easy and convenient. The imaging system and the HDR system are portable and easy moved around. Endo-OCT IGHDR has another advantage in that organ motion will not affect the accuracy of dose delivery similar to EBRT. This is because the radioactive source along with the catheter inside the pancreas would move together with the organ if there is any motion caused by the patient’s breathing or movement.

Further research confirming the ability to accurately detect abnormalities or cancer in the pancreatic duct are needed as well as the ability to safely deliver radiation. Many possible risk factors need to be considered in using the modality, especially the safety of placing the catheter in the pancreatic duct, risks of bleeding, pancreatic duct stricture and pancreatitis (32). To evaluate the safety of the Endo-OCT the next step of our project is to apply this technology to live animals. If this imaging technology is proven effective, it could also be used in diseases of other organs including the lungs, esophagus, and GI tract.

This endoscopic OCT imaging and therapy dual-function system that we proposed and designed accommodates the need to deliver drugs aside from radioactive sources locally to the disease site, albeit it requires further research and financial investment. We have to emphasize that the radioactive sources used in Endo-OCT IGHDR may also be various types of isotopes—this will be determined in our future studies. Our ultimate goal is to apply this patented technology (33) in clinical practice.

## Data Availability Statement

The raw data supporting the conclusions of this article will be made available by the authors, without undue reservation.

## Ethics Statement

The studies involving human participants were reviewed and approved by The Ohio State University IRB#: 2018C0135, OSU18060. The patients/participants provided their written informed consent to participate in this study. Written informed consent was obtained from the individual(s) for the publication of any potentially identifiable images or data included in this article.

## Author Contributions

LL: PI, oversees the design and develops the entire project, participates in clinical trials with the team, operates OCT system, acquires and analyzes images, and writes proposals and manuscripts. ZH: The OCT engineer who designed and built the Endo-OCT system and provided maintenance of the system. Contributed equally to this work with LL. WF: Oversees the portion of pathology study for this project. RS: Performs the clinical trials with the team on resected pancreas and carries out pathologic analysis. WC: Performs the clinical trials with the team on resected pancreas and carries out pathologic analysis. XP: In charge of the statistics analysis in our research project and helped design the clinical trial that was approved by the IRB. Some preliminary results presented in this manuscript are from the IRB approved clinical trial. JG: The radiation oncologist who provides treatment and medical advice on HDR brachytherapy. MB: The GI and pancreatic surgeon and original PI with LL, ZH, and WF who obtained the Pelotonia funding to build the OCT machine and performed the earlier experiments. MD: The GI and pancreatic cancer surgeon and PI to lead the first clinical trial on resected pancreas. All authors contributed to the article and approved the submitted version.

## Funding

This study was supported by (1) Pelotonia Idea Grant 2010 and (2) Grant ID: GR110548 18-1-4 Endoscopic 3-dimensional high resolution optical coherence tomography.

## Conflict of Interest

Author ZH is the founder of company Pharos Scientific LLC and shares the patent with the Ohio State University (OSU) (33). The OSU group has been collaborating with him since the beginning of this research project. 

The remaining authors declare that the research was conducted in the absence of any commercial or financial relationships that could be construed as a potential conflict of interest.

## References

[1] Siegel RL Miller KD Jemal A. Cancer statistics 2020. CA: A Cancer J Clin (2020) 70(1):7–30. 10.3322/caac.21590; Cancer statistics 2018, CA: A Cancer Journal for Clinicians, Vol. 68, Issue 1, January/February 2018, Pages 7-30. 04 January 2018. Available at: 10.3322/caac.21590.

[2] Lee ES Lee JM. Imaging diagnosis of pancreatic cancer: A state-of-the-art review. World J Gastroenterol (2014) 20(24):7864–77. 10.3748/wjg.v20.i24.7864 PMC4069314 24976723

[3] Melo SA Luecke LB Kahlert C Fernandez AF Gammon ST Kaye J. Glypican-1 identifies cancer exosomes and detects early pancreatic cancer. Nature 523:177. 26106858 10.1038/nature14581 PMC4825698

[4] Mellby LD Nyberg AP Johansen JS Wingren C Nordestgaard BG Bojesen SE. Serum Biomarker Signature-Based Liquid Biopsy for Diagnosis of Early-Stage Pancreatic Cancer. J Clin Oncol (2018) 36(28):2887–94. 10.1200/JCO.2017.77.6658 PMC6161836 30106639

[5] Huang D Swanson EA Lin CP Schuman JS Stinson WG Chang W. Optical Coherence tomography. Science (1991) 254(5035):1178–81. 10.1126/science.1957169 PMC4638169 1957169

[6] Fujimoto JG Brezinski ME Tearney GJ Boppart SA Bourna B Hee MR. Optical biopsy and imaging using optical coherence tomography. Nat Med (1995) 1(9):970–2. 10.1038/nm0995-970 7585229

[7] Wojtkowski M Bajraszewski T Gorczyriska I Targowski P Kowalczyk A Wasilewski W. Ophthalmic imaging by spectral optical coherence tomography. Am J Ophthalmol (2004) 138(3):412–9. 10.1016/j.ajo.2004.04.049 15364223

[8] Margolis R Spaide RF. A Pilot Study of Enhanced Depth Imaging Optical Coherence Tomography of the Choroid in Normal Eyes. Am J Ophthalmol (2009) 147(5):811–5. 10.1016/j.ajo.2008.12.008 19232559

[9] Gladkova ND Petrova GA Nikulin NK Radenska-Lopovok SG Snopova LB Chumakov YUP. In vivo optical coherence tomography imaging of human skin: norm and pathology. Skin Res Technol (2000) 6:6–16. 11428936 10.1034/j.1600-0846.2000.006001006.x

[10] Bezerra HG Costa MA Guagliumi G Rollins AM Simon DI. Intracoronary Optical Coherence Tomography: A Comprehensive Review Clinical and Research Applications. JACC: Cardiovasc Interventions (2009) 2(11). 10.1016/j.jcin.2009.06.019 PMC4113036 19926041

[11] Jang I-K ed. Cardiovascular OCT Imaging. Springer (2014). Switzerland: Springer International Publishing.(eBook). 10.1007/978-3-319-10801-8

[12] Stark A Eibl G. Pancreatic Ductal Adenocarcinoma, Pancreapedia: Exocrine Pancreas Knowledge Base (2015) American Pancreatic Association (APA). 10.3998/panc.2015.14

[13] Adamska A Domenichini A Falasca M. Pancreatic Ductal Adenocarcinoma: Current and Evolving Therapies. Int J Mol Sci (2017) 18(7):1338. 10.3390/ijms18071338 PMC5535831 28640192

[14] Isenberg G Pollack MJ Faulx AL Chak A Wong R Farooq F. In Vivo Imaging of the Biliary (BD) and Pancreatic Duct (PD) with Optical Coherence Tomography (OCT) During ERCP Accurately Identifies Dysplastic Cellular Changes. Gastrointest Endoscopy (2008) 67:AB107.

[15] Pelotonia Idea Grant Awards Bloomston MP Lu LC Frankel Hu ZL. Optical Coherence Tomography Imaging of Precancerous Pancreatic Lesion. (2010). Available at: https://cancer.osu.edu/news/pelotonia-idea-grants-to-fund-ohio-state-cancer-research, https://pelotonia.org/the-blog/2010/07/28/idea-grants-awarded/, PELOTONIA11.

[16] Sohn TA Yeo CJ Cameron JL Hruban RH Fukushima N Campbell KA. Intraductal Papillary Mucinous Neoplasms of the Pancreas: an updated experience. Ann Surg (2004) 239:788–99. 10.1097/01.sla.0000128306.90650.aa PMC1356287 15166958

[17] Ryan JF Rosati LM Groot VP Le DT Zheng L Laheru DA. Stereotactic body radiation therapy for palliative management of pancreatic adenocarcinoma in elderly and medically inoperable patients. Oncotarget (2018) 9:16427–36. 10.18632/oncotarget.24713 PMC5893251 29662656

[18] Merchant N Berlin J. Past and Future of Pancreas Cancer: Are We Ready to Move Forward Together? J Clin Oncol (2008) 26:3478–80. 10.1200/JCO.2008.17.0811 18640927

[19] Chakravarthy Abrams R. Radiation Therapy and 5-Fluorouracil in Pancreatic Cancer. *Seminars Radiat Oncol* (1997) 7:291–9. 10.1053/SRAO00700291 10717227

[20] Chang BW Saif MW. Stereotactic Body Radiation Therapy (SBRT) in Pancreatic Cancer: Is It Ready for Prime Time? J Pancreas (2008) 9(6):676–82. 18981547

[21] Koong AC Le QT Ho A Fong B Fisher G Cho C. Phase I study of stereotactic radiosurgery in patients with locally advanced pancreatic cancer. Int J Radiat Oncol Biol Phys (2004) 58:1017–21. 10.1016/j.ijrobp.2003.11.004 15001240

[22] Koong AC Christofferson E Le QT Goodman KA Ho A Kuo T. Phase II study to assess the efficacy of conventionally fractionated radiotherapy followed by a stereotactic radiosurgery boost in patients with locally advanced pancreatic cancer. Int J Radiat Oncol Biol Phys (2005) 63:320–3. 10.1016/j.ijrobp.2005.07.002 16168826

[23] Hoyer M Roed H Sengelov L Traberg A Ohlhuis L Pedersen J. Phase-II study on stereotactic radiotherapy of locally advanced pancreatic carcinoma, Radiotherapy and Oncology. (2005) 76:48–53. 10.1016/j.radonc.2004.12.022 15990186

[24] Qian LW Mizumoto K Urashima T Nagai E Maehara N Sato N. Radiation-induced Increase in Invasive Potential of Human Pancreatic Cancer Cells and Its Blockade by a Matrix Metalloproteinase Inhibitor, CGS27023. Clin Cancer Res (2002) 8:1223–7. 11948136

[25] Vollmer CM Jr Dixon E. Intraductal papillary mucinous neoplasm: Coming of age. World J Gastrointest Surg (2010) 2(10):299–305. 21160834 10.4240/wjgs.v2.i10.299 PMC2999208

[26] Lu LC Hu ZL Frankel W Dillhoff M Grecula JC Bloomston MP. Endoscopic 3-Dimensional OCT-Guided Brachytherapy for Early-Stage Pancreatic Cancers. IJROBP (2016) 96(2 Supplement):S167–8. page A22 (Basic/Translational Science Abstract Award - Radiation Physics).

[27] Kenny KH. Chan and Shuo Tang, High-speed spectral domain optical coherence tomography using non-uniform fast Fourier transform. BioMed Opt Express (2010) 1(5):1309–19. 10.1364/BOE.1.001309 PMC3018116 21258551

[28] Yang MJ Yan ZP Luo JJ Liu Q Zhang W Ma J. A pilot study of intraluminal brachytherapy using 125I seed strand for locally advanced pancreatic ductal adenocarcinoma with obstructive jaundice. Brachytherapy (2016) 15(6):859–64. 10.1016/j.brachy.2016.05.004 27364870

[29] Rey JW Hansen T Dümcke S Tresch A Kramer K Galle PR. Efficacy of SpyGlass^TM^-directed biopsy compared to brush cytology in obtaining adequate tissue for diagnosis in patients with biliary strictures. World J Gastrointest Endosc (2014) 6(4):137–43. 10.4253/wjge.v6.i4.137 PMC3985154 24748921

[30] Krishna SG Modi RM Kamboj AK Swanson BJ Hart PA Dillhoff ME. *In vivo* and *ex vivo* confocal endomicroscopy of pancreatic cystic lesions: A prospective study. World J Gastrointest Endosc (2017) 23(18):3338–48. Published online 2017 May 14. 10.3748/wjg.v23.i18.3338. Cellvizio COOK Medical LLC, Bloomington, IN 47402, USA. Available at https://www.cookmedical.com/products/a2e49b41-c046-4dd1-9427-a55df2371bfb/. 10.3748/wjg.v23.i18.3338 PMC5434441 28566895

[31] Dillhoff ME Lu LC Frankel W Shen R Chen W Pan X. OCT Imaging Clinical Trial on Removed Pancreas with High Risk Pancreatic Cancer. Clin Trial (2018), IRB–OSU-18060.

[32] Bekaii-Saab T El-Rayes B eds. Current and Emerging Therapies in Pancreatic Cancer. Springer (2018). p. 328. ISBN 978-3-319-58255-9 ISBN 978-3-319-58256-6 (eBook). DOI 10.1007/978-3-319-58256-6. Library of Congress Control Number: 2017955090. Chapter 18: Novel Radiotherapy Modalities.

[33] Lu LC Hu ZL. United States Patent Awarded, “Internally-administered Radiation Therapy using Endoscopic Image Guidance. Patent No.: US 10,231,625 B2. Date of Patent: March 19, 2019. Assignee: OHIO STATE INNOVATION FOUNDATION, Columbus, OH (US). Available at: https://patents.google.com/patent/US20160263401A1/en.

