# Supplementary material for: Using Endoscopic Optical Coherence Tomography to Detect and Treat Early-Stage Pancreatic Cancers
Source: Front Oncol. 2021 Mar 15;11:591484. doi: 10.3389/fonc.2021.591484 (PMC8005647; doi:10.3389/fonc.2021.591484)
Supplement: Supplementary file 1 [file DataSheet_1.pdf]

Video Description of the OCT-Guided HDRBRT Working Flow

The following video clips demonstrate the major working flow of our proposed OCT-IGHDRBRT -- from the OCT imaging process to the treatment delivery.

1. **VideoClip1.mp4**

   It demonstrates our first experiment on a resected pancreas from a 76 year old male patient using our OCT imaging device that was built with the support of **Pelotonia Idea Grant Awards (2010)** [Reference 15].

2. **VideoClip2a.mp4, VideoClip2b.mp4, VideoClip2c.mp4**

   These three video clips demonstrate our preliminary experiment step by step that how does the proposed OCT-IGHDRBRT works on a phantom:

   a) **VideoClip2a.mp4** demonstrates how the OCT probe was inserted into the source catheter tube to imaging. The OCT probe moved inside the catheter tube to the region of interested – here it was the center of the tiny hollow cylinder. The tiny hollow cylinder was made from the rolling of two layers Gafchromic film. This hollow cylinder played as a pancreatic duct, which was placed inside a metal phantom that was made of cerrobend alloy block. Note: the catheter tube was inserted into the tiny hollow cylinder before the OCT probe was inserted into the catheter tube.

   b) **VideoClip2b.mp4** demonstrates: (1) the OCT imaging probe was withdrawn back from the imaging region and the outside of the source catheter tube after the imaging process was completed; (2) The OCT catheter tube then was disconnected from the source catheter tube; (3) The source catheter tube was connected to the HDR treatment machine. It was ready to deliver the radioactive source to the center of a tiny hollow cylinder rolled with 2 layers Gafchromic film. Experiment personnel were evacuated from the treatment and the treatment would be started.

   c) **VideoClip2c.mp4** demonstrates HDR brachytherapy: the HDR source was sent to the center of a tiny hollow cylinder rolled with two layers Gafchromic film for the treatment. The treatment was being observed on the monitor at the treatment console area outside the treatment room.
